# Feeling the Squeeze: Nonmarket Institutional Pressures and Firm Nonmarket Strategies

**DOI:** 10.1007/s11575-018-0355-1

**Published:** 2018-07-03

**Authors:** Cosmina Lelia Voinea, Hans van Kranenburg

**Affiliations:** 1 Open University, Heerlen, The Netherlands; 2 Institute for Management Research (IMR), Nijmegen School of Management, Radboud University, Nijmegen, The Netherlands

**Keywords:** Nonmarket institutions, Pressures, Drivers, Transactional and relational strategies and tactics

## Abstract

This study investigates the drivers of pressures from various institutions in the nonmarket environment and the responses of MNEs to these pressures in a host country. By taking a broad institutional perspective, this study pairs and integrates the economic perspective of new institutionalism and the sociological perspective of neo institutionalism with the corporate political strategy perspective. This research provides a systematic review of the drivers underlying pressures from various types of nonmarket institutions that explain the preference of firms to use a transactional or relational strategy to deal with these pressures. The evidence is based on research involving MNEs in the Netherlands. The nonmarket institutions that exert the greatest pressures at the national level pushing MNEs to use transactional more than relational strategies and tactics are regulatory and standards agencies. The pressures of political institutions, interest groups, and the media, in contrast, trigger MNEs to employ relational rather than transactional strategies and tactics.

## Introduction

In the current competitive business landscape, it has become essential for most multinational enterprises (MNEs) not only to focus on the relationships with market actors, but also with nonmarket institutions: Political institutions, regulatory and standards authorities, and social institutions, such as the media and interest groups (Hillman et al. 2004; Mellahi et al. 2016). For many MNEs, these institutions and their power, obligations and influences have a major impact on their sustainable competitive position (Kassinis and Vafeas 2006; Dieleman and Boddewyn 2012; Doh et al. 2012; Lawton et al. 2013). In particular, MNEs with a high dependence on nonmarket institutions may have to pursue political or social objectives in order to align their interests with those of the institutions (Marquis and Qian 2014), establishing the flow of critical resources (Kostka and Zhou 2013) or obtaining the support of critical stakeholders (Wang and Qian 2011). Much MNE research has explored the challenges the organizations face, the strategies and tactics they can undertake in dealing with institutions in various nonmarket contexts and their performance (Kostova and Zaheer 1999; Rajwani and Liedong 2015). For instance, Boddewyn (2016) provides an interesting overview of the studies dealing with the relationships between MNEs and political and regulatory institutions. Marano and Tashman (2011) investigate the relationship between MNEs and nongovernmental organizations. Vachani et al. (2009) show NGO influence on MNE social development strategies in varying nonmarket contexts. However, many studies have investigated a restricted selection of nonmarket institutions in relation to MNE nonmarket behaviour. MNEs must deal with various types of institutions and their pressures in the nonmarket context simultaneously. How they manage the pressures from these institutions depends on the perceived formal and informal power and obligations of the institutions. Nonmarket institutional pressures consist of various drivers. Our knowledge of the composition of drivers and perceived power of nonmarket institutions in a particular setting and the responses of MNEs is still limited (Hiatt et al. 2015). Specifically missing is an examination of how underlying drivers of pressures of various types of institutions affect the nonmarket behaviour of MNEs in a host environment (Lux et al. 2011; Zhang et al. 2016). Doh et al. (2012, p. 23) stress that “both scholars and managers need to understand the institutional factors in more detail in nonmarket research”. Hence, it is imperative to gain a better understanding of the perceived impact of nonmarket institutional pressures on MNEs, the underlying drivers of these pressures and how MNEs behave in a host nonmarket environment.

Therefore, the aim of this study is to explore the various types of nonmarket institutional pressures that influence MNEs nonmarket strategies. We will do so by assessing both the underlying perceived formal and informal drivers of certain classes of nonmarket institutional pressures and the resulting relationships between the classes of pressures and the MNEs’ responses. The extant literature on factors driving institutional pressures has been largely developed from two main perspectives: The institutional perspective and the corporate political strategy perspective. Doh et al. (2012) emphasize that the integration of different perspectives is a logical next step in advancing nonmarket strategy research. Therefore, this study combines institutional and corporate political strategy perspectives to examine the primary drivers of certain classes of institutional pressures facing MNEs in a nonmarket environment and how the organizations respond to these pressures. This study further contributes to the nonmarket strategy literature because we investigate the relationships between various types of nonmarket institutional pressures and the nonmarket behavior of MNEs in a non-US setting.

Aguinis and Glavas (2012) emphasize that institutional theory can help elucidate why MNEs may feel compelled to develop nonmarket activities and strategies. Ioannou and Serafeim (2012) stress that institutional theory provides insight into the challenges facing MNEs in establishing legitimacy in various host nonmarket contexts. According to Doh et al. (2012), nonmarket strategy research is embedded in multiple institutional perspectives and levels of analysis. Two important perspectives in institutional theory relevant to the field of nonmarket strategy and MNEs are new institutional economics (Clougherty and Grajek 2008) and neo-institutional perspective (Orr and Scott 2008). Therefore, this research applies the economic perspective of new institutionalism and the sociological perspective of neo institutionalism, because their overall orientation emanates from similar traditions, level of analysis, and perspectives (Doh et al. 2012). Both perspectives emphasize the importance of political, social and economic institutions in constraining and facilitating the nonmarket behavior of firms.

Taking this broad institutional perspective, this study also incorporates a corporate political strategy perspective to investigate strategic responses to various nonmarket institutional pressures. The corporate political literature defines and measures different types of reactive and proactive nonmarket strategies (e.g., Baron and Diermeier 2007; Blumentritt and Nigh 2002; Boddewyn and Brewer 1994; Meznar and Nigh 1995). One commonly used typology was developed by Hillman and Hitt (1999) (Hillman et al. 2004; van Kranenburg et al. 2017; Mellahi et al. 2016). They classified the types of nonmarket strategies into two broad categories. Firms can develop relational nonmarket strategies that are long-term oriented and create a certain in-depth base within the nonmarket environment meant to avoid or decrease nonmarket influences on their activities. They can also develop and implement a transactional nonmarket strategy to deal with nonmarket actors and issues in their environment. This type of strategy is based on mainly event-specificity and temporary actions. Firms can use both types of strategies simultaneously to respond to nonmarket institutional pressures (Nell et al. 2015).

Notwithstanding the growth of nonmarket strategy studies, Lawton et al. (2013) emphasize that studies in the nonmarket strategy field have traditionally focused on the United States, where particular nonmarket strategies are used and are considered more ethically appropriate than in other developed countries. The reason for the US focus is that hard data and ample material are available to develop and test hypotheses. Many emerging and developed economies do not have the same degree of transparency as the United States. In addition, many developed economies have more collegial firm nonmarket institutions relations and are less conflicting than those in the US. The United States can be characterized as a pluralist economy. Nonetheless, a growing interest exists in expanding nonmarket strategy research beyond the US domain. In addition, much of this work analyzes the firm-political institution relations, but does not include various nonmarket institutions (Peng et al. 2009; Boddewyn 2016). The empirical evidence of this study comes from MNEs operating in a corporatist economy the Netherlands. Therefore, a second contribution of this study to the nonmarket strategy field is the analysis of how firms perceive the drivers of pressures of nonmarket institutions in a non-US setting. The Netherlands is a small, industrialized economy with an open integrated economy and one of the founding members of the European Union (EU). There is strong and close cooperation amongst employers’ organisations, labour unions and the government, leading to abundant negotiations, as well as substantial rules and regulations that oversee an extensive welfare state. This close cooperation has led to both a stable economic and political environment and to joint initiatives for economic integration in Europe that have made the Netherlands an interesting host location for MNEs. The Netherlands represents one of the largest recipients of foreign investments in the world and, due to its favorable location and active role within the European Union, many MNEs have chosen the country as strategic orientation (UNCTAD 2011). Data on foreign firms were obtained through a questionnaire survey and existing data sets.

Results show that the factors that determine the significant pressure of regulatory agencies mainly involve a temporary issue perspective on rules and regulations, insufficient autonomy of regulatory institutions and insufficient transparency in terms of rules and regulations. With regard to standards agencies, the pressure stems from costs, both in terms of complying with imposed standards and in terms of obtaining permits, licenses and authorisations. The institutional drivers that are specific to the pressure of political institutions include the saliency of policy issues and the costs of politics. Interest groups exert pressure through their ability to influence public opinion and through their collective concerns. In addition, institutional drivers related to media pressures include media credibility and its societal influence. Furthermore, evidence shows that the pressures from political institutions, interest groups, and the media are more likely to elicit relational rather than transactional strategies and tactics, whilst pressures from regulatory agencies, and standards agencies engender transactional nonmarket more than relational strategies and related tactics.

The structure of this study is as follows: Sect. 2 explores the drivers of pressures from various types of nonmarket institutions that trigger different nonmarket strategies of firms, and presents the hypotheses. In Sect. 3, we present the study sample, method and data, along with a structural equations model. Section 4 contains the analysis and empirical results; we conclude with a discussion of the results and implications in Sect. 5.

## Hypotheses

### Institutional Environment

Institutional theory has gained momentum in exploring nonmarket strategy research (Henisz and Delios 2004). This theory has a rich and diverse set of traditions with different perspectives on the relationship between actors and institutions. Two dominant institutional perspectives that have gained traction in the nonmarket strategy literature are the new institutional economics (North 1990) and neo institutionalism (DiMaggio and Powell 1983). Although these perspectives have offered varied conceptualization of institutions and their impact, the perspectives are not discrete or discriminant (Doh et al. 2012). Hence, pairing and integrating these perspectives will advance the nonmarket strategy field. New institutional economics is an economic perspective that focuses on the social and legal norms and rules that underlie economic activities. It studies the role that culture, legal systems, and political institutions have on economic development. North (1990) defines institutions as the rules of the game in a society and the humanly devised constraints that shape human interaction and structure political, economic and social interactions. Such constraints are devised as formal rules (constitutions, laws, property rights) and as informal restraints (sanctions, taboos, customs, traditions, codes of conduct), which usually contribute to the perpetuation of order and safety within a market or society. Informal constraints are more a result of tradition while formal institutions serve to solve the problem of trust and protection (Mantzavinos 2001). Here institutions have a largely constraining character, setting clear boundaries on actor behavior (Ingram and Clary 2000). Institutions provide structure and order by aligning actor actions and expectations. Although institutions are seen as exogenous to actors, they can change over time. According to North (1990), it is the interaction between institutions and organizations that shapes the institutional evolution of the economy. Therefore, to understand an institution, it is important to look at both the rules and the actors, seeing each as interdependent of the other.

The second dominant institutional perspective in the nonmarket strategy field is neo institutionalism. This sociological perspective on institutionalism emphasizes the social structures and relationships that occur within society and how these structures define and shape broader systems and the role of organizations within them (Doh et al. 2012). While the new institutional economics perspective stresses that people consciously design institutions to help them efficiently meet their goals, neo institutionalism stresses that institutions are not human designs but rather evolve from the particularities of a given historical and cultural context (Dimaggio and Powell 1983). Barley and Tolbert (1997, p. 98) therefore define institutions as “historical accretions of past practices and understandings that set conditions on actions”. The construction of an institution, commonly referred to as the process of institutionalization, occurs through collective political, social and cultural acceptance of practices driven by the attainment of legitimacy for these actions. Scott (2008) gives a less deterministic interpretation of institutions. He focuses more on inter-subjectivity and individual interpretations rather than large social processes. Scott (2013, p. 56) defines institutions as “comprised of regulative, normative and cultural-cognitive elements that, together with associated activities and resources, provide stability and meaning to social life”. Accordingly, institutions are categorized into three pillars: The regulative, normative and cognitive pillars. In particular, institutions based on the regulatory pillar have the ability to establish rules, monitor compliance with these rules, and deliver sanctions, rewards or punishment when necessary to influence future behavior (Scott 2013). These regulative activities can either be enforced formally by government authority or can be enforced more informally through societal pressure. Normative systems constrain social behavior through rights, responsibilities, privileges, duties, mandates and licenses (Geels 2004; Scott 2013). The social prescriptions that define legitimate behavior, transmitted for instance by the state or professional associations, may become taken-for-granted over time and, as a consequence, are very difficult to change (Suchman 1995).

Hence, neo institutionalism is concerned with the social context within which organizations operate and as such seeks to understand social structures which have attained a high degree of resilience (Scott 2013). These social structures have often existed for decades and have only been subjected to small changes over time (Van den Hoed and Vergragt 2006). As a consequence, institutions often create stability, as well as inertia, in a social system. Thus, the main premise of the sociological perspective on institutionalism holds that organizations are influenced by institutional logics (Greenwood et al. 2011). It suggests that firm behavior is a direct reflection of the degree of conformity of organizations to the institutional pressures imposed on them by their environment. However, it is evident that in reality, firms can and will not uniformly respond to institutional pressures, as their behavior as a response to these pressures will vary depending on the context and nature of the institutional pressures they are confronted with (Oliver 1991; Greenwood et al. 2008). In other words, “organizational behavior may vary from passive conformity to active resistance in response to institutional pressures, depending on the nature and context of the pressures themselves” (Oliver 1991, p. 146). Thus, taking a broader institutional perspective is valuable in understanding organizational behavior as a response to the environment. A main premise of institutional theory is that the institutional environment and the resulting institutional pressures have a profound influence on organizational behavior, often more substantial than market pressures (Meyer and Rowan 1977).

### Nonmarket Strategies

Firms should go beyond formulating market strategies and thoroughly consider complementary strategies to encounter complex influences outside the market, and to increase their legitimacy, performance and ultimately their competitive position. These nonmarket strategies involve actions carried out in social, political and legal arenas (Baron 1995; Shaffer and Hillman 2000) to counter pressures from various nonmarket institutions. Firms can proactively participate in the nonmarket environment to achieve their objectives and potential benefits from nonmarket behavior (Baysinger 1984). These proactive nonmarket strategies and tactics complement market strategies and related tactics, monitor public interests in the nonmarket environment and further assist firms in coping with interdependence issues between market and the nonmarket environment, influencing their market activities (Baron and Diermeier 2007; Boddewyn 2003; Doh and Lucea 2013; Shaffer and Hillman 2000).

Different strands of strategy research have examined the strategies that firms employ to manage the nonmarket environment (Mellahi et al. 2016). Organization response perspective (Oliver 1991) and the corporate political strategy (Hillman and Hitt 1999) perspectives are often used in the nonmarket strategy research. Research in the field of corporate political strategy focuses on the rationale for firms to have a corporate political strategy, the characteristics of firms engaging in corporate political activities, and to what extent firm’s strategies and related tactics are effective. Based on nonmarket and corporate political strategy literature, proactive nonmarket strategies can be regarded as transactional and relational strategies (Hillman and Hitt 1999). Relational strategies and tactics are defined as proactive practices which minimise surprises from political institutions, regulatory and standards agencies and social institutions such as interest groups and the media, and exercise control over institutional processes, aiming to maximise the alignment of the firm with its environment and with collective interests (Mahon et al. 2004). The relational repertoire includes long-term oriented actions and cooperative tactics meant to strategically interact and to pursue strategic goals through social and political leverage (Hillman and Hitt 1999). Furthermore, relational strategies and tactics serve to build relationship networks with various actors active in the nonmarket environment in order to leverage social capital. Relational strategies and tactics help firms to build critical mass vis-a-vis nonmarket issues that affect the organisation or to build a certain reputation (Baron and Diermeier 2007; Itoh 1993; Keim and Zeithaml 1986) to increase mutual trust and information transfer and to improve joint problem-solving (Hillman and Hitt 1999; Keim and Zeithaml 1986). Such collaborations not only bring mutual benefits from knowledge transfer between organisations, but they also facilitate the creation of new knowledge and produce synergistic solutions (Hardy et al. 2003). Moreover, relational strategies and tactics such as collective networks, business associations’ participation, and stakeholder cooperation enable firms to anticipate possible future issues or changes in the nonmarket environment, detecting a potential nonmarket threat or opportunity. While relational strategies anticipate firms’ future needs and plans, transactional strategies emphasize more urgent needs over planning for the future (Hiatt et al. 2015). Transactional strategies and tactics are represented as issue-specific and reactive deeds of a non-repetitive nature. They are characterised by arm’s-length ties and are undertaken on an ad-hoc basis (Mahon et al. 2004), thus leading to self-interest motivated conduct without network building purposes (Kaufmann 1998; Uzzi 1997).

This strategy type represents a reactive response to changes in the nonmarket environment, undertaken only when the management is forced to act due to visible effects on the firm. Reacting to things that happen, rather than making things happen, is generally characteristic of transactional strategies and tactics. Transactional nonmarket strategies and tactics may include issue-lobbying, temporary grassroots mobilisation of employees, suppliers or customers, advocacy advertising, contracting media experts, press releases and press conferences. The resources for such strategies and tactics are mobilised provisionally to deal with a certain event or to accomplish a specific (image or reputation) target (Hillman and Hitt 1999). Transactional strategies and tactics are often used by firms to fine-tune or complement relational ones. Additionally, in particular situations, when it is not possible to use relational strategies and tactics due to legislation and regulation, then firms can only rely on transactional actions (van Kranenburg et al. 2012). Relational and transactional nonmarket strategies can be concurrently implemented; thus, firms can make intertwined and simultaneous use of these two broad types of nonmarket strategies (Hillman and Hitt 1999). Both types of nonmarket strategies consist of the information, financial incentive, and constituency-building strategies and their related tactics (such as lobbying, campaign contributions, grassroots mobilization etc.) or a configuration of these strategies and tactics, although the form, intensity, and frequency of use differ between both types of nonmarket strategies (Hillman and Hitt 1999; Holburn and Van den Bergh 2004; Keim and Zeithaml 1986; Schuler 1996; Schuler et al. 2002).

### Nonmarket Institutions

A plethora of nonmarket institutions influence firms formally, through laws and regulation, and informally, through social pressure, activism and efforts to shape the public perception of business. Political institutions, regulatory and standards agencies, and social institutions such as the media and interest groups have emerged, each unravelling a different societal need and resolving political, social, or economic issues. The pressures of these institutions consist of different drivers. The response of firms to an institutional pressure depends on how these firms perceive the impact, power and importance of these drivers. The following section outlines the main drivers of each institutional pressure and how firms respond to these pressures.

### Political Institutions

Political institutions are organizations which create, enforce, and apply laws; that mediate conflict; make (governmental) policy on the economy and social systems; and otherwise provide representation for the populous. All formal and informal provisions, rules and norms that guide the political decision-making process together form the system of political institutions. Political institutions are reflections of a nation’s culture, its aspirations, and its history, and these institutions also play a role in shaping government’s policies. The term political institutions may also refer to the recognized structure of rules and principles within which firms operate, including such concepts as the right to vote, responsible government, and accountability. These institutions, formal and informal, determine both the constraints and incentives faced by key actors in a given society (Börner 2005). Political institutions have the power to affect the legal and economic environment (Shenkar and Luo 2008). They can enact and enforce laws thereby influencing the legal environment and with the coercive power they can let firms adopt certain practices or policies. They also set monetary and tax policies, price controls, and intellectual property regulations and they also influence labor relations, trade policies, capital and exchange controls, and transfer pricing policies (Shenkar and Luo 2008). Moreover, political institutions efficiently reduce transaction costs in the political processes. Given the endogenous feature of political institutions and the strategic allocation of powers they provide, appropriately chosen institutions can help the development of credible mechanisms capable of decreasing risks of opportunistic behavior of political and economic actors (Börner 2005).

Political institutions thus also represent nonmarket arrangements, which offer instances of public authority and public policy related issues, costs and benefits, affecting firms that participate in the political arena (Choi et al. 2014; Weingast 1995). Political institutions are closely associated with forms of government (Persson 2002) and politics may be attractive for firms, provided that the political arena grants opportunities for achieving favourable policy outcomes (Bonardi et al. 2005; Grossman and Helpman 1996). In general, firms tend to get involved in the political arena when policy outcomes affect their businesses or in order to obtain beneficial policy outcomes (Masters and Keim 1985). Similarly to economic markets, for which industry attractiveness justifies the entry of firms, firms get involved in the political nonmarket arena and interact with political institutions when policies are attractive or when they may affect their business operations (Choi et al. 2014). The political pressure results from the salience of political issues and the costs and benefits of engaging in politics (Bikhchandani et al. 1992).

Saliency refers to public awareness of a specific issue in which firms also have an interest (Bonardi et al. 2006). If the issues for which firms seek to obtain favourable policy outcomes have little salience (low public interest), firms enjoy a relatively strong advantage in shaping final decisions (McCubbins et al. 1987). These greater chances for success in their political endeavours increase their nonmarket political incentives.

Efforts to develop strategies to mitigate policies that might develop into a salient issue also offer venues for nonmarket action (Hillman and Keim 1995; Laffont 1996). For example, responding to an issue before it becomes politicised or widely salient may enable the firm to resolve that issue to its benefit, depending on its life cycle (Bigelow et al. 1993). Firms that delay their responses can lose substantial decision-making discretion, because over time, legislation gets crafted and regulations enforced. Because policy issues also derive from firms’ agendas, firms can best advance policy through regular interactions with institutions and the building of long-term relationship networks (Choi et al. 2014), hiring people with government experience or who have held political positions in governmental bodies, as well as people who have made other contributions (Dean and Brown 1995) outside the market (Baum et al. 2000; Benton and Daly 1991; Buchanan 1980). Such efforts, on the part of firms, in obtaining desirable policy outcomes represent the costs of politics or political involvement (Laffont 1996). If desired policy outcomes can be achieved with diffused costs and then can lead to concentrated benefits, firms will find politics and political actions more attractive (Glynn and Abzug 2002).

Hence, decisions and activities of political institutions have real economic implications for firms. They establish the rules of economic exchange, make particular actions subject to penalties, and can increase the cost of business activities (Hiatt et al. 2015). The nonmarket strategy literature has generally asserted that firms become political active for the purpose of obtaining favourable public policies (White et al. 2017). Bushman et al. (2004) find that firms are more transparent and share information in economies with lower state influence and strong political institutions. Higher transparency and more sharing of information reduce the cost of business activities. Building a strong network with political institutions helps firms to increase the transparency and to share information. In addition, this network can provide firms with unique information about political processes that are often difficult and expensive to obtain (Frynas et al. 2006). A strong network of relations with political institutions also helps firms to find and develop political resources and capabilities to deal with issues and also to find patterns of behaviour in a specific environment (Bonardi et al. 2006; Lawton et al. 2013). Obtaining political capital enables firms to be more effective in the political and social processes and may ultimately lead to improved performance. For instance, White et al. (2017) stress that there is a relationship between firm’s perception of the impact of pressures from political institutions and the intensification of political ties. Ties are characterized by the degree of frequency of interaction between partners and their time and resource commitment (Rowley et al. 2000). Strong ties involve more time and resource commitment and have higher frequency of interactions than weak ties. Weak ties are cheaper to form and easier to maintain than stronger ties. However, strong ties enhance trust, the sharing of more and specific knowledge and information, mutual gain, reciprocity, and a long-term perspective (Larson 1992). These strong ties with political institutions provide firms with a more in-depth understanding of pending political events or social impact, and may develop goodwill (Doh et al. 2012). Given the importance of the salience of political issues, the costs of politics and to obtain favourable public policy, firms’ nonmarket actions focus mainly on relational strategies and tactics. This leads us to formulate the following hypothesis:

*Hypothesis 1a: The pressure of political institutions is manifested through the salience of political issues and the costs of politics.*

*Hypothesis 1b: The pressure of political institutions positively contributes to firms’ use of more relational strategies than transactional strategies.*

### Regulatory Agencies

With the purpose of protecting society from market failures such as monopolies and concentrations, some regulations are needed for the market environment (Ogus 2002). These market failures are not solved by private law and therefore political institutions need to set the rules of the game. They can develop laws and regulations. Regulations, on the other hand, are standards and rules adopted by administrative agencies that govern how laws will be enforced. Regulations can be divided into two types: Social and economic (Ogus 2002; Voinea and van Kranenburg 2017). The goal of social regulations is consumer protection, environmental protection, health and safety. Regulatory actions such as information disclosure, mandatory standards and licensing are important instruments to secure these social goals. Economic regulation, in contrast, is needed when there is insufficient or unfair competition. It consists of legal measures that control, for instance, the price and quality of products and services (Ogus 2002).

Regulatory agencies are public establishments that exercise autonomous statutory authority over specific areas of activities, with a regulatory or supervisory capacity (Scott 2013). They perform their functions with oversight from the legislative part of the executive branch of the government. They cover administrative law and rulemaking codifying and enforcing rules, imposing supervision or oversight. Some regulatory agencies also perform audits, while others are authorised to impose punitive measures on relevant parties (Kanter 1999). Regulatory agencies commonly oversee the use of public goods, ensuring the fair distribution of social welfare, or regulating activities of organisations, institutions, and firms (North 1990). These agencies operate independently from other branches or arms of the government. They can develop policies to achieve their goals and tasks. These policies may differ in the timing of their effectiveness (Motta 1994). An important factor for successful social and economic regulations and policies are regulatory agencies that have a reputation of acting fairly (Stern 1997). Effective regulatory agencies provide transparency and predictability. The effectiveness of regulatory agencies is positively related to the degree of independence of these agencies from their external environment (Voinea and van Kranenburg 2017). Hence, to serve the regulatory objective, regulatory agencies should offer perfect transparency, enjoy complete autonomy in their regulatory progression and practice and engage in consistent proceedings both over time and designated locations (Lewis and Sappington 1991). These traits all have positive connotations, though in practice, some deficiencies in transparency, autonomy and consistency characterise virtually all regulatory agencies (Keim and Zeithaml 1986; Lewis and Sappington 1991). Insufficient transparency would lead to information asymmetries or to knowledge gaps related to policy outcomes with strong implications for firms’ operations (Holburn and Van den Bergh 2004). Through relational actions (i.e. networks or business associations) firms can build social capital, decreasing the information gap and liability caused by the imperfect transparency in regulatory processes (Zaheer 1995). Furthermore, through collective networks firms can also achieve information transfers and joint problem-solving arrangements (Hillman and Hitt 1999; Keim and Baysinger 1988).

When national regulatory institutions lack complete autonomy for establishing, promulgating and implementing rules and regulations, a heritage of state intervention, government policy, or excessive regulations persists, heavily constraining firms’ activities (Stern and Holder 1999; Suchman 1995). Remnant policies influence the autonomy of regulatory agencies and in particular the operation of effective courts, which would enforce proper, consistent rules and regulatory procedures (Stern 1997). Insufficient autonomy is also manifest, as pervasiveness or abuses of power, defined as the firms’ likelihood to encounter abuses of power during normal interactions within actors in market and nonmarket environment (Bardhan 1997; Treisman 2000). The degree to which abuses of power are regular, significantly influences economic and non-economic firm activities, creating opportunities to internalise environmental threats through absorption (Ring et al. 1990) or to purchase facilitating services and favourable regulatory decisions (Ang and Cummings 1997; Boddewyn and Brewer 1994). For firms, managing the abuse of power (or mitigating its effects) represents a nonmarket target, achieved through various nonmarket relational actions, such as increasing interactions and collaboration with local and national actors in order to decrease the pervasiveness of abuse in transactions (Rodriguez et al. 2006).

A kind of mutual dependency relationship exists between regulatory agencies and the firms they regulate. Regulatory agencies must base their decisions on information, but most of the information is confidential and firm-specific. These agencies partly depend on the information provided by firms to make legitimate and acceptable decisions. Therefore, providing specific information is the most tactical strategy regulated firms can use while trying to influence regulatory agencies (Holburn and Van den Bergh 2008).

The pressure of regulatory agencies increases when regulatory actions and promulgations reflect a temporary issue-related perspective. A temporary issue perspective increases the complexity of the regulatory framework and makes it more difficult for firms to grasp future developments. Relational nonmarket strategies and tactics might increase in response to the possible repercussions of a temporarily issue regulatory outlook, such that firms might hire specialists to gain insight into certain regulatory aspects and awareness of regulatory processes. Hence, managing the pressure exerted by regulatory agencies, firms can use relational nonmarket actions (e.g., collective networks, business association participation, partnerships with likeminded firms), in order to compensate for their information gaps and other liabilities given by promulgations with a temporarily issue perspective, by insufficient transparency or by the lack of autonomy of regulators (Boddewyn and Brewer 1994; Hillman and Hitt 1999; Hillman et al. 2004; Mahon et al. 2004). Given the importance of independence, fairness and transparency of these agencies, they are generally reluctant to have long-term relationships with firms. Moreover, given the focus of regulatory agencies on administration, monitoring, and enforcement, firms’ nonmarket actions focus mainly on transactional strategies and tactics. Therefore, we hypothesise that:

*Hypothesis 2a: The pressure of regulatory agencies is explained by insufficient transparency, insufficient autonomy and temporarily issue perspectives on rules and regulations.*

*Hypothesis 2b: The pressure of regulatory agencies positively contributes to firms’ use of more transactional actions than relational actions.*

### Standards Agencies

Whereas regulatory agencies establish rules and objectives for various groups, a specific group of agencies act as mediators, implementing the regulatory requirements. These standards agencies mainly focus on the social goal. Regulatory means such as information disclosure, mandatory standards and licensing are important instruments to secure these social goals. Thus, in addition to regulatory pressures, firms must acquiesce to standards, obtain licenses and obey principles or customs imposed in the business setting (McCubbins et al. 1987). Therefore, standards agencies become important nonmarket institutions, functioning to ensure social loss abatement or loss abatement for firms (Palmer et al. 1993). Social loss abatement entails minimising firms’ activities, if they generate health or safety hazards that are detrimental to the public interest. It also implies optimising the degree of loss imposed by the administrative or other compliance costs associated with regulations (Ogus 2002).

Standards agencies establish and define measures to be taken by firms, before they may enter regulated business arenas. They also frame firms’ activities, through prior approval requirements, mandatory standards and information disclosures (input or output prohibitions) (Rao et al. 2001). Information disclosure requirements demand that firms reveal adequate information regarding their quality and safety practices, in order to help customers exercise their preferences. Information disclosure also helps to avoid the welfare losses that result when consumers are deprived of choice (Shaffer 1995). Firms might be allowed to select the manner in which they disclose information, as long as it matches the imposed standards of the institutional setting (Lenway and Murtha 1994).

Prior approval, in contrast, obliges firms to obtain a license or permit from the authorised institutions before they may lawfully engage in an activity or supply a product or service. To obtain such authorisation, firms usually must fulfil optimal loss abatement requirements or meet other conditions that imply extra costs (Ang and Cummings 1997; Meznar and Nigh 1995). The costs to comply with standards and obtain permits and licenses are the direct results of imposed criteria; they are often high or seem to represent unjustified standards (De Soto 2000; Ogus 2002). Expenditures for fulfilling these requirements thus appear peripheral or groundless to firms and, in response, firms may seek to decrease what they perceive to be unnecessary expenditures through transactional nonmarket actions, such as lobbying for the abrogation of standards (De Soto 2000; Oliver 1991) or temporary mobilisation of employees, suppliers and customers; advocacy advertising; public relations and press conferences (Hillman and Hitt 1999). Firms might try to identify, educate and motivate action groups or stakeholders affected by the same norms. Furthermore, given the focus of standards agencies on administration and enforcement firms' nonmarket actions focus on contesting rules and requirements, disguising nonconformity and damage control (Oliver 1991). In this sense, the pressures from standards agencies, manifested through additional organisational costs to meet various requirements should increase nonmarket transactional actions of the firm and we therefore hypothesise:

*Hypothesis 3a: The pressure of standards agencies is manifested through the costs to obtain permits and licenses and the costs to comply with imposed standards.*

*Hypothesis 3b: The pressure of standards agencies positively contributes to firms’ use of more transactional actions than relational actions.*

### Interest Groups

Interest groups are organised collections of individuals motivated by social and ethical concerns which aim to advance a broad set of interests. Interest groups seek to influence business practices, firm and industry practices, as well as political and economic decisions (Ades and DiTella 1999; Baron 1999; Pacheco et al. 2014). Interest groups are thus collections of individuals that engage in collective action to achieve some desirable end that they could not attain by acting alone (Teegen et al. 2004). They have great loyalty to certain ideals (their reason for existence), and they actively voice these ideals to among other firms, governments, policy-makers, politicians, and media in order to influence firm- or business practices and policy discourses in line with the groups’ demands and ideals (Teegen et al. 2004; Wapner 1995).

Interest groups can influence the perception or assumption of whether the actions of a firm are desirable or appropriate according to a socially constructed system of norms, value, beliefs, and definitions within a society. For instance, interest groups can erode a firm’s market value, destroy its brand, destabilize employee morale, constrain its influence with various constituencies and limit its scope for strategic action (Yaziji and Doh 2009). A wide variety of active interest groups focus predominantly on post-material, nonmarket issues, such as consumer concerns, environmentalism and minority rights, rather than on economic issues, such as import duties, human resource training, or farm price support (Haveman 1993; Pacheco et al. 2014). Generally, interest groups start social movements and, as a result, achieve their goals by gaining public support (Bonardi et al. 2006; Dasgupta et al. 1979). Their capacity to influence public opinion is their greatest power and the primary indicator of the amount of pressure the group is able to exert on decision makers. The extent to which an interest group can make a specific matter salient is referred to as saliency capacity (Buchanan 1980; Vachani et al. 2009).

Interest groups express collective concerns and interests and have a broad influence, shaping social, economic and political systems to promote a given set of values and ideas, whereas the economic advocacy activities of interest groups are relevant for the business environment (Chandler 2013; Mahon et al. 2004; Teegen 2003). Interest group advocacy on economic issues may result in a change in public attitudes. For example, what was acceptable corporate behavior in the past, may no longer be acceptable now and what is acceptable now may not be in the future.

To manage pressures from interest groups, firms try to incorporate issues of concern into their long-term mission statements or transform their (core) values to reach collective goals, as well as implementing other activities or programs aimed at better aligning firm activities with the collective concerns of interest groups (Landes and Posner 1975; Pacheco et al. 2014).

Recent decades, the influence of interest groups on the legitimacy and performance of firms has increased. The rising influence of interest groups can be explained by increasing social and political freedom, the emergence of the welfare state, the shift in social systems of values and societal conceptions, and by developments in information technology (Yaziji and Doh 2009). Nongovernmental organizations (NGOs) are a special type of interest group. The 1970s and 1980s were witness to an explosive growth in the number of nongovernmental organizations (NGOs), yet not until the 1990s did the organizations become publicly recognized as a sector rather than individual activist organizations (Marberg et al. 2016). Different interest groups operate under different ideologies and will have different targets. These different ideologies, norms and values result in the use of various influence tactics to support interest group claims towards firms. For instance, NGOs can use a confrontational approach or an engaging approach (Winston 2002). NGOs which use the first approach tend to employ moral stigmatization, or ‘naming and shaming’, as their primary tactic while NGOs that favor the engaging approach offer dialogue in order to persuade firms by means of ethical and prudential arguments to adopt voluntary codes of conduct and limited forms of cooperation (Voinea and van Kranenburg 2017).

As a consequence of these developments, firms capitalise on building a strong reputation and a sense of being legitimate, by managing the normative pressures imposed by interest groups (Campbell 2007; Chandler 2013; Vachani et al. 2009), by adapting to the guidelines proposed by interest groups (Bonardi and Keim 2005), as well as by monitoring specific business functions to achieve a socially responsible reputation (Baron and Diermeier 2007; Vachani et al. 2009). Given the fact that interest groups focus on nonmarket issue identification and saliency development, firms prefer more to use nonmarket relational strategies to shape collective values and criteria, and to balance expectations (Teegen 2003). Therefore, we advance the following hypothesis:

*Hypothesis 4a: The level of pressure exerted by interest groups on firms is determined by their capacity to gain favourable public opinion and economic advocacy concerns.*

*Hypothesis 4b: The pressure of interest groups positively relate to firms’ use of more relational actions than transactional strategies.*

### Media

Another social nonmarket institution that is becoming of growing importance to firms is the media (Asp 2014; Hennig-Thurau et al. 2010; Kaplan and Haenlein 2010; Voinea and van Kranenburg 2017). This institution consists of multiple mediums, which refers to any means, agency or instrument of communication (Danesi 2000). The media encompass all goal-oriented technical means or instruments for the procurement of information in print, visual, or auditory forms as well as the organizational and institutional entities behind them that generate and provide this information. The information is directed at a broad and public audience (Wirtz et al. 2011). The role of the media can be divided into three core parts (Asp 2014; Baron and Diermeier 2007; Hunter et al. 2013). Firstly, the media act as powerful independent institutions constraining actions and shaping both individual and organizational behavior (Asp 2014). Secondly, the media in their dependent role alert the public, activist, interest groups and government on issues that concern firms’ business activities. Thirdly, the media are used by other institutions such as the government, activists and interest groups to alert the public of firms misdoings. Hence, the media alert the public, activists, interest groups and government of market and nonmarket issues, raising concerns about firms’ practices and reducing the costs of collective nonmarket actions, through their influence and power over public opinion or society at large (Bardoel and d’Haenens 2004). The media continuously shape public opinion and have a central role in educating, informing and empowering the public, regarding social responsibility and sustainable development (Shaffer and Hillman 2000). As a nonmarket institution, the media can also influence organisational activities by affecting firms’ reputation and ethical status, and by placing firms’ activities under the scrutiny of other market and nonmarket institutions.

Although the media can significantly influence firm activity, their roles are neither fixed nor well-defined. The roles are not only determined, implemented and interpreted by the owners and managers, but also by legislature, government administrative agencies, judicial institutions, public sentiment and ethical consensus. Furthermore, the legitimacy claim of the media is of mixed nature, as the media can take on a dependent role as well as an independent role (Asp 2014; Fassin 2009). Therefore, credibility is the central determinant of the media’s influence on business and society (Bucy 2003), stemming from perceptions of media ‘believability’, accuracy, bias, fairness and information completeness (Johnson and Kaye 1998). The concern for the public interest stems from the media’s role in terms of informing the public and uncovering malpractice and corruption at all levels of society, holding governments and businesses accountable for their actions, related to both market and nonmarket issues (Bardoel and d’Haenens 2004; Landes and Posner 1975). For firms, the media can also amplify or reduce uncertainty by providing information, presenting facts and events, changes in legislation, exploring their potential significance and ramifications and advocating possible courses of actions (Pfeffer and Salancik 1978).

The media are considered one of the primary sources for the general public to learn about firms beyond those that they have relationships with (Carroll 2009; Deephouse 2000). Media inform the public about issues and present an overall evaluation of the firm (Deephouse 2000). Newsworthiness of a firm’s activities along with its performance, the diversity of its responses, its communication effort, time, and memory decay determine the firm’s image and thus its reputation (Garbett 1988). Due to the media’s pervasiveness and agenda-setting power, the media can play a significant role in a firm’s reputation. Studies show a relationship between media exposure and firm’s reputation (Wartick 1992). The effect of media exposure on reputation depends on situational factors such as source credibility and firm’s responses (Griffin et al. 1991). For instance, the media generally cover issues of firms of great social significance, characterised by great public interest and concern, such as malpractice and unethical business behaviour. The potential for media coverage puts pressure on firms to achieve more open, transparent business practices, as well as to address their ethics of their nonmarket behaviour, with respect to business practices and stakeholders (Bardhan 1997). Media coverage can also contribute to firms’ awareness of the likelihood of encountering corruption in normal interactions (Bardhan 1997; Treisman 2000), and the inherent degree of ambiguity associated with transactions. When the media have high credibility and strong societal influence, firms make use of this to build relationship networks with the media, in order to improve their image and reputation. Firms can also use regular press releases and media interviews to present their views and motivations, casting issues in particular light. This kind of framing can also shape audiences’ assessment of a firm’s reputation and practices (Bansal and Clelland 2004). However, frames are not fixed and issues can be reframed over time. Bach and Blake (2016, p. 66) stress that “framing is a powerful strategic tool that enables firms to shape the structure of the nonmarket environment to their advantage”.

In recent years, due to information and communication technological developments, the traditional and new media have become integrated into a contemporary socially-networked multi-channel platform. As a result, the new media landscape has become more complex and uncertain, speed of sharing information has increased, and controlling the information sharing process has become more difficult (Kaplan and Haenlein 2010). Building a network with various media organizations can help firms to deal with the new situation and develop the needed nonmarket capital. Firms should build ongoing relationships with journalists and media sources.

Furthermore, a mutual resource dependency relationship exists between media and firms (van Kranenburg 2017). As Picard (2013, p. 49) pointed out, “the fundamental problem for news providers is that news itself has never been financially viable as a market-based good. It has always been primarily financed by arrangements based on income derived from sources other than selling news to consumers”. Therefore, many media firms are operating in two markets: Readers and advertisers markets. Media organizations depend on the advertising income derived from firms. They need to ensure that there is an audience for their advertisers. Hence, they need to focus on news values and market driven values such as profitability and maximizing readership. Therefore, media organizations also prefer a long-term relationship with firms in order to secure access to financial sources. Thus, firms exploit media credibility and its influence for their organisational benefit (reputation building), through different relational strategies and tactics, such as building long term relationships with media institutions. Therefore:

*Hypothesis 5a: The pressure of the media is determined by its high credibility, high societal influence and concern for public interest.*

*Hypothesis 5b: The pressure of the media is positively related to firms’ use of more relational actions than transactional strategies.*

## Methodology

### Context, Sample and Data

An empirical study was conducted amongst managing directors of multinational corporations operating in the Netherlands, by means of a postal questionnaire survey. As it is a small, industrialised country with an open, integrated economy and a founding member of the European Union (EU), the Netherlands embraces the Polder Model, which seeks consensus policies in economics, consensus in decision making, pragmatic recognition of pluriformity and cooperation, despite the differences between various actors. Specifically, the Polder Model focuses on tripartite cooperation amongst employers’ organisations, labour unions and the government, leading to lengthy negotiations, as well as a host of rules and regulations characteristic of a welfare state. Hence, the Dutch economy has the characteristics of both a regulated market economy with a large social security system and a shareholder capitalism economy aimed at maximizing profits, although the Rhineland model still dominates. The Netherlands can be described as a corporatist country.

Because the Netherlands was among the countries that initiated the European Customs Union in 1957, the economy has also benefited from the early establishment of substantial economic integration. Its membership in the EU helps counteract the adverse effects of its small size by extending its domestic market. The Netherlands has become one of the largest recipients of foreign direct investment (FDI) in the world, and with its favourable location and active role within the EU, foreign firms choose it strategically as a host country. In 2012, foreign MNEs delivered a turnover of €366 billion, representing 30% of the total turnover in the Netherlands. Furthermore, the sample population accounted for 21% of overall investments and for 22% of the investments in research and development (Netherlands Foreign Investment Agency 2012).

The Netherlands is home to some strong competitive sectors internationally, in particular food and beverage, petroleum and chemicals, and transportation, although it also has robust finance, insurance, and service sectors. The most important service sectors include business services (a very broad category covering employment agencies, translators, cleaning and real estate for business, but also the distribution of electricity, water, gas and other oil products), construction services (which includes not only house construction, but also infrastructure and dredging), and postal services (Hogenbirk et al. 2009). The political and economic climate in the Netherlands makes it an interesting case to explore how firms perceive the pressures from various national institutions and their drivers and how they respond to these pressures. It is important to note that political campaign contributions are prohibited in the Netherlands. Therefore, firms can only use the information and the consistency-building strategies and their related tactics.

Several databases were used for this study: The Dun and Bradstreet database, Osiris, Political Constraint Index Dataset (Henisz 2011) and the Dow Douglas Psychic Distance Stimuli (Dow Douglas 2012). In addition, we collected data by using a postal survey sent to among managing directors of foreign firms in the Netherlands during the summer of 2011. In total, 800 multinational corporations were selected from the Dun and Bradstreet (2010) database. To improve our survey and to address the non-response bias, we also conducted 17 in-depth interviews with firms’ managers and discussed the survey items and tested for non-response bias. It is important to understand the effects of non-response bias because bias jeopardizes the accuracy of estimates derived from surveys and thus the ability to draw inferences about a general population from the sample (Sax et al. 2003). To test for non-response bias we employed two techniques: (a) We compared early and late returns but spotted no significant differences in responses regarding key constructs; (b) we used the so called non-response follow up; that is, we systematically contacted respondents either by phone and inquired about main constructs or through face-to-face meetings and in-depth interviews. Through interviews we used participant comments and suggestions to revise the survey and assess the non-response bias. The information collected from the non-respondents through the follow-up technique did not differ significantly from the respondents. Subsequently, we carried out a pilot survey to evaluate the revised survey instrument.

The returned responses totalled 160 foreign MNEs (25% of the sample group) operating in the Netherlands and originating from 22 home countries: Austria, Belgium, Bermuda, Canada, Denmark, England, Finland, France, Germany, India, Ireland, Italy, Japan, Luxembourg, Norway, Netherlands, Portugal, South Africa, Spain, Sweden, Switzerland, and the United States. In addition, we also collected data among managing directors of domestic firms during the spring of 2012. The questionnaire was sent to 400 medium and large domestic companies in the Netherlands. After sending two reminders, the total number of returned responses was 40.

### Method

Due to the novelty and interpretation flexibility of the nonmarket field, nonmarket variables can be measured through different manifest indicators; structural equation modelling (SEM) is an appropriate method for the field. Furthermore, we used partial least squares path modelling (PLS), as implemented in SmartPLS 3.0, to assess the reliability and validity of measurement, to estimate the model coefficients and to test the hypotheses. It is a widely accepted variance-based, descriptive and prediction-oriented approach to structural equation modelling and can be applied for exploratory and confirmatory research (Chin et al. 2003; Hair et al. 2012). A PLS path model is composed of a structural component specifying the relationships between latent variables and a measurement part, specifying the relationships between latent variables and their observed or manifest variables. PLS focuses on maximising the dependent variables’ variance that the independent variables can explain. Furthermore, as PLS accommodates models combining formative and reflective constructs, it is recommended for analysing small to medium size samples.

### Measurement

All exogenous constructs are operationalised by means of formative multi-item scales. All indicators were measured on a 5-point rating scale, with '1' representing the lowest level and '5' the highest. Based on the literature, we used two items to measure the *pressure of political institutions*: Salience of political issues and the costs of political involvement (Bikhchandani et al. 1992; Bonardi et al. 2006). Three items were used to measure the exogenous variable *pressure of regulatory agencies*: Insufficient transparency of rules and regulations, insufficient autonomy of regulatory institutions and short-term perspectives on rules and regulations (Bardhan 1997; Stern and Holder 1999; Suchman 1995; Treisman 2000).

To measure *pressure of standards agencies*, we used the costs for obtaining permits, licenses and authorisations and the costs to comply with standards (Ogus 2002; Rao et al. 2001). For the construct *pressure of interest groups*, we used economic advocacy concerns and capacity to influence public opinion (Bonardi et al. 2006; Buchanan 1980; Vachani et al. 2009). Regarding *pressure of the media*, we measured credibility, societal influence and concern for public interest (Bardoel and d’Haenens 2004; Bucy 2003; Haley 1996).

The *endogenous construct nonmarket strategies* involves reflective measurement. It is constructed from the questionnaire survey. Survey questions measured whether each firm uses a transactional or relational nonmarket strategy. We used the information, financial incentive, and constituency-building strategies and their related tactics defined by Hillman and Hitt (1999) to select the items. Based on a 5-point scale ranging from 'never used' (indicating various possible actions specific to transactional behavior) to 'continuously used' tactics, specific to relational strategies (Nell et al. 2015). The inquiring tactics are as follows: Business associations’ participation; partnerships to influence political issues; interest groups’ awareness and networks; lobbying; supply of technical reports to regulators; press releases; testimonies in expert hearings; contributions to political parties; presence of firm’s members in political parties; employing people with political experience; employee training for trade union participation; employee training for media relations; development of an internal prevention system; development of an internal monitoring system; proactive self-changing and regulation; building socially responsible reputations; challenging government regulations; interaction with local governments; interaction with national government; active cooperation with Dutch institutions; ad-hoc cooperation with Dutch institutions; having a clearly established set of tactics to deal with Dutch institutions; providing local or national government with products or services. The frequency of use determines whether the item is part of relational or transactional strategies. Although the types of proactive nonmarket strategies are classified into two broad categories, the use of one type of nonmarket strategy does not preclude the use of another. Firms can use both types of strategies simultaneously (Hillman and Hitt 1999).

### Controls

#### Firm Level

The resources available to respond to nonmarket issues may also influence the type of nonmarket strategies (Hillman et al. 2004; Lawton et al. 2013). In addition, firms with a large resource and employment base have more assets at risk, which means that they might be more affected by changes related to legislation, regulations and standards (Masters and Keim 1985). Moreover, large firms are generally more interdependent in terms of a variety of institutions (Meznar and Nigh 1995). We measured *size* as the number of employees of each firm in the Netherlands.

Furthermore, a firm’s experience with institutions is considered an indication of possible network and social capital. More experience may facilitate certain types of nonmarket strategies, including building networks or cooperation with local and national governments (Hillman and Hitt 1999; Keim and Baysinger 1988; Keim and Zeithaml 1986). Since the experience of a firm might relate to the type of nonmarket strategy, we considered it as a control variable, measured by the *age* of the firm.

#### Industry Level

Institutional factors specific to regulatory agencies ensure reforms in the regulated industries in which firms operate (Keim and Baysinger 1988). Regulatory agencies also ensure third-party control in some industries, to avoid or penalise anti-competition behaviour. The relational and transactional nonmarket strategies undertaken by firms can thus differ across industries and we therefore controlled for possible industry effects with *industry dummies: Manufacturing*, *Services* and *Utilities*. The category *Manufacturing* includes the following industries: Chemical products; extraction and processing of non-energy minerals (2.5% of the final sample); metal manufacturing; mechanical, electrical and instrument engineering; office and data process machinery (43% of the final sample); manufacturing of food, drinks and tobacco; textiles; clothing; paper; rubber and plastic (18.0% of the final sample). The category *Services* includes retail and distribution; hotels; catering; repairs (6.5% of the final sample); transport and communication (9% of the final sample); banking; finance; insurance; business services (consultancies, PR and advertising) (13% of the final sample). The category *Utilities* includes energy and water (8% of the final sample).

#### Country Level

To explore institutional pressures more broadly and to expand the range of institutional issues, we included in our model a broader designation of institutional pressures that is, socio-cultural institutional pressures and political system pressures. MNEs from particular contexts have more experience or are used to particular types of nonmarket strategies. Therefore, the variables *socio*-*cultural pressures* and *political system pressures* incorporate possible country effects on the type of nonmarket strategy used by firms. The variable socio-cultural pressures captures the difference between the home and host country-designated socio-cultural factors, which affect the flows of information between firm and environment; this is measured as the difference between home and host country index estimates, provided by Dow and Karunaratna (2006).

The variable political system pressures measures the difference between home and host political constraints and identifies the difference between the underlying political structures, measuring their ability to support credible policy commitments. The primary source for these estimates was provided by the Political Constraint Index Dataset by Henisz (2011).

## Results

### Evaluation of the Measurement Model

**Fig. 1:**
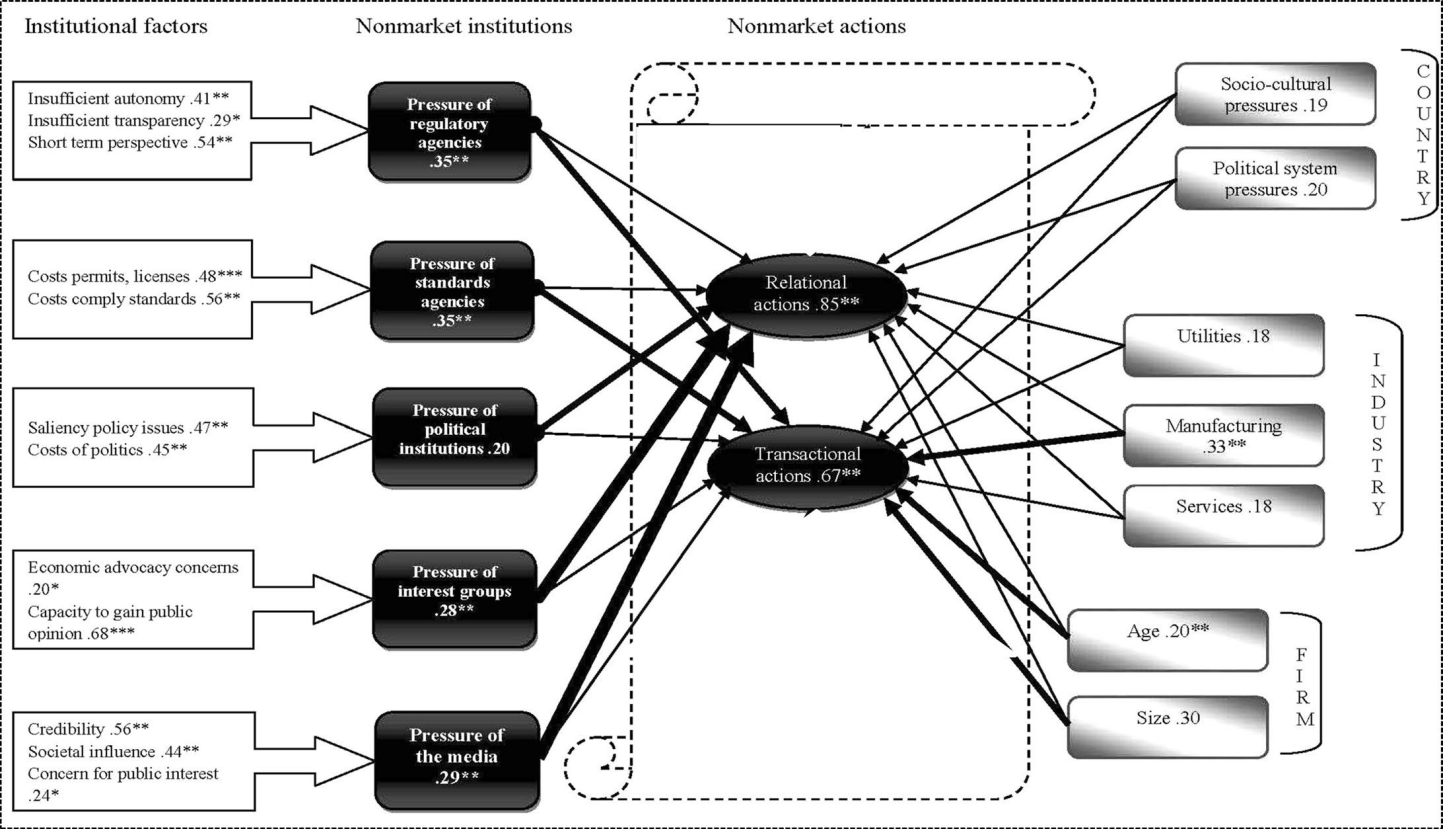
The structural model and results. *Significant 0.1 level; **significant at the 0.05 level; ***significant at 0.01 level; bold arrow=highest explanatory value; medium arrow=medium explanatory value; line arrow=least explanatory value

The causal and empirical measurement results are presented in Fig. 1. The central criterion for evaluating the SEM was the rate of reliability (R^2^) of the latent endogenous variable nonmarket strategies; the value of 0.74 suggests the robustness of the measurement model, i.e. the nonmarket institutional effects explain 74% of the variance of the latent endogenous variable. The five latent exogenous variables in our SEM result from formative measurement models, including respective factors and items pertaining to each institution that explain its pressure, as visible in Fig. 1.

According to Chin and Newsted (1999) the evaluation criteria for the manifest indicators are the weights of 0.02, 0.05 and 0.35. If such weights are achieved, then we can conclude that the latent variables have small, medium and large effect sizes, respectively. The evaluation of the empirical results, regarding the formative measurement model, reveals that the theoretically deduced manifest indicators are very well suited for the latent variables (Diamantopoulos and Winkelhofer 2001). Therefore, we analyse first the factors that are underlying the pressures from the various institutions and the composition of the nonmarket strategies.

The manifest indicator ‘saliency of policy issues’ offered the highest value (coefficient=0.47; t-value=1.98) in explaining the *pressure of political institutions*, followed by costs of politics (coefficient=0.45; t-value=1.96). The R-square of pressure of political institutions equalled 0.74, suggesting that the manifest indicators offer a good measurement model for this construct. Thus, we can accept hypothesis H1a. The R-square of the exogenous construct *pressure of regulatory agencies* reached 0.70, in support of the robustness of the measurement model for this construct. The manifest indicator for the ‘short-term perspective of rules and regulations’ offered the greatest explanatory value (coefficient 0.54; t-value=1.96) regarding the pressure of regulatory agencies, followed by the item ‘insufficient autonomy of regulatory institutions’ (coefficient=0.41; t-value=2.00) and ‘insufficient transparency of rules and regulations’ (coefficient=0.29; t-value=1.96); therefore, H2a is accepted.

To explain the *pressure of standards agencies*, the item ‘costs to comply with imposed standards’ achieved the highest value (coefficient=0.56; t-value=2.00), followed by the manifest indicator ‘costs to obtain permits, licenses and authorizations’ (coefficient=0.48; t-value=1.96). The R-square of this exogenous variable exhibited a value of 0.72, confirming the robustness of the measurement model; thus, H3a can be accepted. *Pressure of interest groups* also obtained a robust measurement model (R-square=0.74), explained mainly by the item ‘capacity to gain public opinion’, with its strong explanatory power (coefficient=0.68; t-value =2.00). The other indicator, ‘economic advocacy concerns’, exhibited a lower weight of only 0.20 (t-value=1.96); nonetheless, H4a can also be accepted. The exogenous variable *pressure of the media* was mostly explained by the manifest indicator ‘credibility’, with the highest value of 0.56 (t-value=1.96), followed by ‘societal influence’ (coefficient=0.44; t-value=1.96). The item ‘concern for public interest’ (coefficient=0.24; t-value=1.94) had the smallest explanatory value for this latent construct. The R-square reached 0.68, suggesting that the manifest indicators create a good measurement model for this variable and that H5a can be accepted.

### Evaluation of the Structural Model

The measures of both endogenous and exogenous constructs suggest that our data are robust. Furthermore, the correlation statistics between the constructs fall within acceptable ranges (Fornell and Bookstein 1982). In Table 1 we present the correlations of the constructs, along with construct-level measurement statistics. The results show that the correlations were low for all variables, so multicollinearity was not a concern.

**Table 1:** Construct-level measurement statistics and correlation of constructs

Variables	1.	2.	3.	4.	5.	6.	7.	8.	9.	10.	11.	12.
1. Regulatory agencies	1.00											
2. Standards agencies	0.18	1.00										
3. Political institutions	0.27	0.25	1.00									
4. Interest groups	0.16	0.14	0.79	1.00								
5. Media	0.24	0.28	0.29	0.39	1.00							
6. Age	0.27	0.23	0.24	0.45	0.23	1.00						
7. Size	0.14	0.34	0.26	0.36	0.21	0.28	1.00					
8. Utilities	0.21	0.19	0.31	0.42	0.34	0.34	0.25	1.00				
9. Manufacturing	0.24	0.27	0.21	0.27	0.23	0.26	0.34	0.32	1.00			
10. Services	0.20	0.28	0.26	0.32	0.36	0.28	0.32	0.20	0.24	1.00		
11. Socio cultural pressures	0.24	0.31	0.17	0.14	0.24	0.20	0.36	0.32	0.31	0.24	1.00	
12. Political system pressures	0.14	0.22	(0.62)	0.31	0.22	0.25	0.37	0.32	0.22	0.20	0.35	1.00

Numbers on the diagonal shown in bold denote the square root of the average variance extracted

Raw measures mean-centered prior to creating interactions

Correlations greater than an absolute 0.50 are shown in parentheses

Table 2 presents the significance of the interrelations between the manifest and latent variables, after using a bootstrapping procedure, which is appropriate for determining the significance of the interrelations between the latent endogenous and latent exogenous variables (Bollen and Stine 1993; Efron and Tibshirani 1993).

**Table 2:** Results of bootstrapping procedure

Variables	Beta-coefficients	T-values
Regulatory agencies	0.35**	2.00
Standards agencies	0.35**	2.02
Political institutions	0.20	1.29
Interest groups	0.28**	1.96
The media	0.29**	1.96
Age	0.20**	2.00
Size	0.30	1.96
Utilities	0.18	1.28
Manufacturing	0.33**	1.96
Services	0.18	1.28
Socio cultural pressures	0.19	1.35
Political system pressures	0.30	1.30
Relational strategies	0.85**	1.96
Transactional strategies	0.67**	1.95

*Significant 0.1 level; **significant at the 0.05 level

The explanatory value of the relationship between the pressure of political institutions and firms’ relational actions is relatively low (t-value=1.29; coefficient 0.20); therefore, H1b is rejected. The pressure of regulatory agencies has a significant, positive effect on the increase in nonmarket transactional strategies, rather than relational ones undertaken by firms, with a t-value of 2.00 (coefficient 0.35); therefore, H2b is accepted. Empirical estimates also show that the pressure of standards agencies exerted a significant effect (t-value=2.02; coefficient 0.35), in terms of increasing nonmarket transactional strategies, therefore we accept H3b.

Further empirical tests offered significant explanatory value regarding the pressure of interest groups; there was an increase in nonmarket relational strategies undertaken by firms (t-value=1.96; coefficient 0.28), as determined by the predicted manifest indicators. Therefore, we can accept H4b. Finally, with regard to the institutional pressure of the media, we found that its manifest variables and results indicated significant explanatory value for increased nonmarket relational strategies (t-value=1.96; coefficient 0.29), therefore we accept H5b.

Regarding the control variables, the age (coefficient=0.20; t-value=2.00) and size (coefficient=0.30; t-value=1.96) of the firm had a medium level explanatory power in terms of the increase in transactional nonmarket strategies. Moreover, some variance in nonmarket actions is attributed to the type of industry. The explanatory value of firms in the manufacturing industry (t-value=1.95; coefficient 0.33) is significant. These firms exhibit an increase in transactional strategies, when dealing with institutional influences. With regard to the country level variables, empirical testing showed that the difference between home and host country social cultural and political designated institutional factors have no significant influence on the type of nonmarket strategies.

## Discussion and Conclusions

Much more than background conditions, nonmarket “institutions determine what arrows a MNE has in its quiver as it struggles to formulate and implement nonmarket strategy, and to create competitive advantage” (Ingram and Silverman 2002, p. 20). Political institutions, regulatory and standards agencies, and social institutions, such as the media and interest groups have progressively emerged, each responding to different societal needs or responding to different market and nonmarket issues. MNEs must align with the host institutional environment in order to manage political, regulatory social pressures and priorities rather than transplant their home nonmarket practices within their network of subsidiaries (Aquilera-Caracuel et al. 2012). Despite managerial and academic acknowledgment of the importance of nonmarket institutions, complex aspects of the pressures of nonmarket institutions, their drivers and their impacts on MNE behaviour remain ambiguous (Dean and Brown 1995; Doh and Lucea 2013). How MNEs deal with the pressures imposed by nonmarket institutions depend on the perceived formal and informal power and obligations of these institutions. Many previous studies have focused on one or a restricted number of nonmarket institutions, but have not included the main institutions that MNEs deal with in their host nonmarket environment. In addition, many of these studies in the nonmarket strategy field have traditionally focused on the US where the institutional context is different than in other developed countries. Our study is one of first to investigate simultaneously how MNEs perceive the pressures created by various nonmarket institutions in relation to the MNEs nonmarket behavior, and to empirically test in a non-US setting the main drivers of the pressures imposed by these institutions. The data from this study is from the Netherlands, a small open industrialized economy. It can be characterized as a corporatist country.

The extant literature on factors driving institutional pressures and organizational responses have been largely developed from different strands of literature. Doh et al. (2012) and Mellahi et al. (2016) provide interesting overviews of nonmarket strategy literature in the international business field. Lawton et al. (2013) and Boddewyn (2016) provide overviews of the development of corporate political research in the international business. However, these excellent studies do not provide an overview of the main nonmarket institutions and the drivers that create pressures on MNEs and how MNEs deal with these pressures. Through our study, we attempt to shed light on important drivers which elucidate the pressures that nonmarket institutions can exert on firms and influence their nonmarket responses. Whilst it is difficult to delimitate the drivers, nonetheless the first step is to analyze whether a particular institution (outside the market span) still exerts pressure or influence on a firm’s activities and triggers an organizational response. If such pressure or influence and respective organizational response are supported by empirical evidence, we can then include such particular institution under the range of nonmarket institutions.

This study used two dominant, distinct but related institutional perspectives: The economic perspective of new institutionalism and the sociological perspective of neo institutionalism. These perspectives are generally not combined in the nonmarket strategy field. Doh et al. (2012) stress that integration of institutional and strategic perspectives provides a logical path to advance nonmarket strategy research. Peng et al. (2009) also emphasize the importance of including the institutional perspective in studies about firm strategies in the international business field. Therefore, by taking a broad institutional perspective, this study paired and integrated the economic perspective of new institutionalism and the sociological perspective of neo institutionalism with the of corporate political strategy perspective, contributing to the nonmarket strategy research. We used the corporate political literature to define and measure firm nonmarket responses. Our justification for this is that the seminal work of Hillman and Hitt (1999) provides an excellent instrument to define nonmarket strategies and their related tactics. Hence, this study investigated the composition of underlying drivers of pressures of various political, regulatory and social institutions in a particular host environment and the preference of firms to use transactional or relational strategies–that is the frequency of use of tactics related to the specific information, financial incentive, and constituency-building strategies.

Our evidence shows that the pressure of regulatory agencies explained by an ad-hoc and temporary issue perspective on rules and regulations and insufficient transparency in terms of rules and regulations increase nonmarket transactional actions. Firms carry out on an ad-hoc basis interactions with governmental agencies and regulators, given the fact that representatives change constantly and no window of opportunity for relationship building is offered (van Kranenburg et al. 2012). Results regarding standards agencies influencing firm activities through costs to comply with imposed standards and to obtain permits, licenses and authorisations show that standard agencies normalise business activities and commonly enforce standards and safety. The vast array of required permits and licenses restrict and set boundaries for business practices and activities. When these costs seem groundless or unnecessarily high, firms intensify their nonmarket transactional activities in an attempt to reduce them. Furthermore, the pressure of political institutions, explained through the saliency of policy issues and the costs of politics only slightly relate to triggering nonmarket relational strategies and tactics. Again, this finding might be explained by a characteristic of the environment: in the Netherlands, political involvement on behalf of firms is not common practice, nor can policy issue agendas be set easily by businesses (van Kranenburg et al. 2012). Also, as a nonmarket institution, interest groups exert influence through their ability to influence public opinion and their economic advocacy concerns, triggering from firms an increase in nonmarket relational actions. Thus, firms increase the frequency of their nonmarket relational actions to monitor their business functions and augment their accountability within society, in an effort to build a reputation for responsibility (Baron and Diermeier 2007).

Because the media can make or break the image of a firm by empowering the public with information about social duties, sustainable growth and various firm positions on these matters, firms tend to increase their nonmarket relational actions to manage the pressures of the media (Hillman and Hitt 1999; Shaffer and Hillman 2000).

Furthermore, an interesting finding is that MNEs with a different background based on political and social cultural system do seem not to differ in their type of response to institutional pressures in a corporatist country like the Netherlands. However, MNEs with more experience with the nonmarket context prefer to use more transactional strategies than relational ones.

This study shows that domestic firms and MNEs operating in the corporatist country the Netherlands both perceived a significant impact of the pressures of the main nonmarket institutions on their operations. They respond to these pressures with both transactional and/or relational strategies. MNEs employ these proactive strategies simultaneously, although the intensity of use of these tactics differs between firms. The intensity of use depends on the age of firms. MNEs considering establishing a presence in the Netherlands should begin by implementing relational strategies in order to respond to nonmarket pressures. Doing so will likely increase their credibility and reputation (van Kranenburg et al. 2017). In addition, becoming acquainted with the Dutch context and its profusion of bureaucratic rules and regulations also is necessary as such regulations may limit how newly established MNEs learn about and respond to nonmarket institutional pressures in the Netherlands. It would be interesting to determine whether the drivers of pressures are perceived similarly in other corporatist countries, or whether our findings are specific to the Dutch context.

Overall, our study elucidates firms’ managerial proceedings by clarifying which institutions are most relevant and can exert regulatory, political or social pressures on business activities. Context-specific influences demand consideration in the course of strategic choices about the institutional environment, especially if the aim is to develop social capital, establish networks and increase responsiveness to—or become embedded in—the country’s business setting (Schuler 1996; Shaver 1998; Yoffie and Bergenstein 1985). Also, there is a need for theoretical awareness, in terms of the fact that not all institutions act as facilitators for the overall business environment. Contrary to market institutions, which facilitate business activities, nonmarket institutions, which create interdependencies between the market and nonmarket arena, may hinder business activities and thus justify the implementation of nonmarket actions.

With our work, we have taken a step further towards developing a robust overview of drivers associated with nonmarket institutional pressures that can explain why firms undertake nonmarket actions. This brings us closer to the ‘why’ and ‘how’ of firms respond to these pressures. Due to their conflicting demands and interests, understanding nonmarket institutional pressures, in relation to firm behaviour, requires an inclusive approach, which simultaneously considers various types of institutions.

### Limitations and Implications

Our study has a number of limitations that should be considered beyond the need to understand the underlying drivers of pressures of institutions in the nonmarket environment and the strategic responses of firms. First, our study remains exploratory in nature, particularly regarding the development of the notion of nonmarket. While we effectively measure multiple pressures that the firm is exposed to, our constructs cannot capture the precise content of the underlying drivers of pressures. Due to the rapid development of social media, we were not able to precisely measure the drivers and the impact of the pressure of the new media. At the time the survey was conducted, firms were not fully aware of the impact of new media on their behaviour. Our formative indicators offer glimpses into the relative importance of items that form the constructs, and these results lay significant groundwork for further investigations, deterministic procedures and analyses of similar data. In addition, our data about impact of underlying drivers of pressures are based on information from single respondents, leading to rather subjective evaluations. Furthermore, the data reflect the perceptions of the MNEs in one country. In terms of further research, we recommend including more countries in order to gain deeper insight into the various types of nonmarket institutions which influence firms’ actions in different nonmarket contexts.

This study did not include the interaction between market and nonmarket environment. The nonmarket strategy does not stand alone, but it should contribute to the success of the market strategy of the firms and ultimately the performance and sustainable competitive advantage of firms (Baysinger 1984; Hillman and Hitt 1999; Hillman et al. 2004; van Kranenburg and Voinea 2017; Mellahi et al. 2016; Sun et al. 2012). Studies in the international business context also suggest that the socially responsible behavior of MNEs can support nonmarket strategies and tactics, in particular the corporate political activities of these firms, to strengthen their legitimacy and facilitate the nonmarket tactics and strategies to deal with institutional pressures (e.g., Jamali and Karam 2016; Joutsenvirta and Vaara 2015; Marquis and Qian 2014). Therefore, further nonmarket strategy research should include corporate social responsibility and the market environment of firms. Additionally, a mixed nonmarket-market model could simultaneously include economic and non-economic determinants to extend beyond traditional market strategies and advance a new concept, incorporating an inclusive range of constituents.

### Further Research

This research is embedded in the economic perspective of new institutionalism and the sociological perspective of institutionalism. Building on these two perspectives, we recognize a third relevant institutional perspective that is likely to advance the nonmarket strategy research and provide insights into this field (Doh et al. 2012; Nell et al. 2015). The third perspective is the national business system which is also connected to the other two perspectives. The national business system refers to country specific systems for corporate governance, systems of collective bargaining and relations with stakeholders. Pairing and integrating this perspective with new institutional economics and neo institutionalism would provide a useful organizing framework for understanding the range of nonmarket strategy perspectives and would advance nonmarket strategy research. It focuses on more attention to institutional diversity and the role of actors. This would allow future research to include two theoretical effects: The country of origin effect in accordance to which MNEs’ strategies and practices are shaped by systems of the country of origin such as corporate governance systems and systems of collective bargaining; and the dominant effect, which includes the idea that MNEs from dominant countries of origin are able to transfer organizational practices across countries (Dekocker et al. 2012). Furthermore, we are aware that currently many economies (also the Netherlands) are regionally integrated; this plays an important role in the institutional setting and factors as well as in the institutional interplay and inter-influences. Lawton et al. (2013) pointed out that the political, regulatory and social arrangements are converging in the European Union. For instance, the national business systems of member states have become more integrated. The role of the European Commission has become more dominant; it increasingly orchestrates the processes between the member states, industries and firms. Therefore, a valuable direction for future research is given by the vertical institutional integration in the field of nonmarket strategy research, to include supranational institutions and their influence on firm nonmarket actions. Thus, nonmarket institutional pressures could also include the interplay between national and supranational institutions, offering a multitude of political, cultural, social and economic potential issues (Aghion and Tirole 1997; Hillman 2003; Kostova and Zaheer 1999; Zaheer 1995; Zaheer and Zaheer 1997). National-level institutions fall within supranational arrangements of various types; for every issue area (regulatory, political and social), various combinations of supranational and intergovernmental elements govern the transactions (Moravcsik 1991). Thus, nonmarket institutional pressures, stemming from a supranational echelon, could imply the existence of vertical nonmarket institutional pressures. Further investigation of these nonmarket institutional pressures would bring us a step closer to framing this challenging concept.

The resource dependency theory posits that MNEs can use particular activities to increase legitimacy and performance to influence the dependence of MNEs on institutions in the nonmarket environment (Getz 2001). In the context of the nonmarket strategy literature, the resource dependence theory is often used in conjunction with stakeholder or institutional theory. For instance, Kassinis and Vafeas (2006) argue that while stakeholder theory explains why and how stakeholders influence firm conduct and performance, the resource dependence theory provides insight into the ability of stakeholders to influence MNE decisions. Arya and Zhang (2009) stress the importance of a firm’s stakeholder influence capacity to identify, act on and profit from opportunities to improve stakeholder relationships. These theories contribute to the understanding of how firm resources and capabilities are integrated, reconfigurated and deployed in the context of different nonmarket institutions. This study complements earlier research which has used various institutional perspectives to explain the drivers of pressures of nonmarket institutions and the nonmarket strategies of firms. This study did not address the needed resources and capabilities and the managerial decision making and coordinative processes and capabilities by which firms assemble and leverage resources and capabilities to develop nonmarket strategies. Building on these theories might be beneficial in moving towards a more integrated approach that links the different levels of analysis between organizational and institutional contexts. Consequently, future research could develop an assessment of power based on specific nonmarket resources and capabilities and the control of these resources and capabilities by the firm and its stakeholders. Another benefit of further exploring the relationships between firms and various nonmarket institutions in a more integrated approach would be determining which resources and capabilities are required to deal with the political, regulatory and social pressures from the institutions and to increase the performance of firms. Due to scarcity and cost of resources and development of capabilities, it is important that firms learn how to allocate the resources and capabilities to market and nonmarket activities given their financial and other constraints. Building on these ideas is likely to advance nonmarket strategy research and provide further insights into this field of study.
